# Supplementary figures and images for: Collective Animal Behavior from Bayesian Estimation and Probability Matching
Source: PLoS Comput Biol. 2011 Nov 17;7(11):e1002282. doi: 10.1371/journal.pcbi.1002282 (PMC3219619; doi:10.1371/journal.pcbi.1002282)

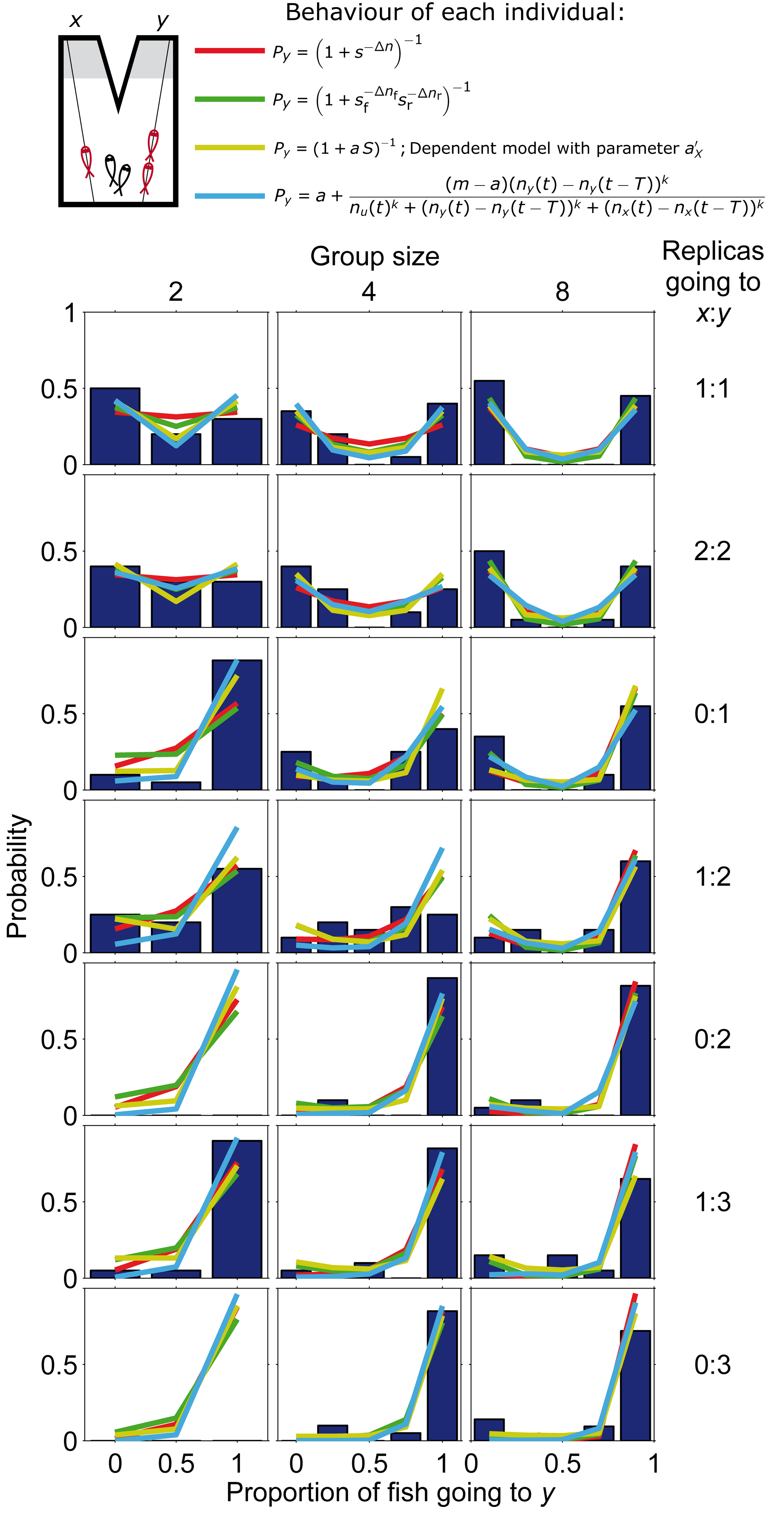

Supplement: Figure S1 — Comparison between different models for the symmetric set-up. Experimentally measured statistics of final configurations of fish choices from 20 experimental repetitions [42] (blue histograms). Red line: results from our single-parameter model assuming independence in Eq. 17 in the main text (). Green line: Enhanced model assuming independence with different reliability for the replicas (, ). Yellow line: Model including dependencies (). Blue line: Empirical model presented in Ref. [42], using the parameters reported there. Different graphs correspond to different stickleback group sizes and different number of replicas going to and . According to Bayesian Information Criterion (BIC, see Methods), the best model is our model with dependencies (yellow line, logprob , and BIC weight . Second-best is the complicated version of the model without dependencies (green line, logprob , and BIC weight ). Third-best is our one-parameter model assuming independence (red line, , ). And last (but not far from the third one) the model from Ref. [42] (blue line, ). For the model from Ref. [42], and correspond to a re-optimization of the model as described in Methods, because using the parameters reported in [42] would perform worse). (TIF) [file pcbi.1002282.s001.tif]

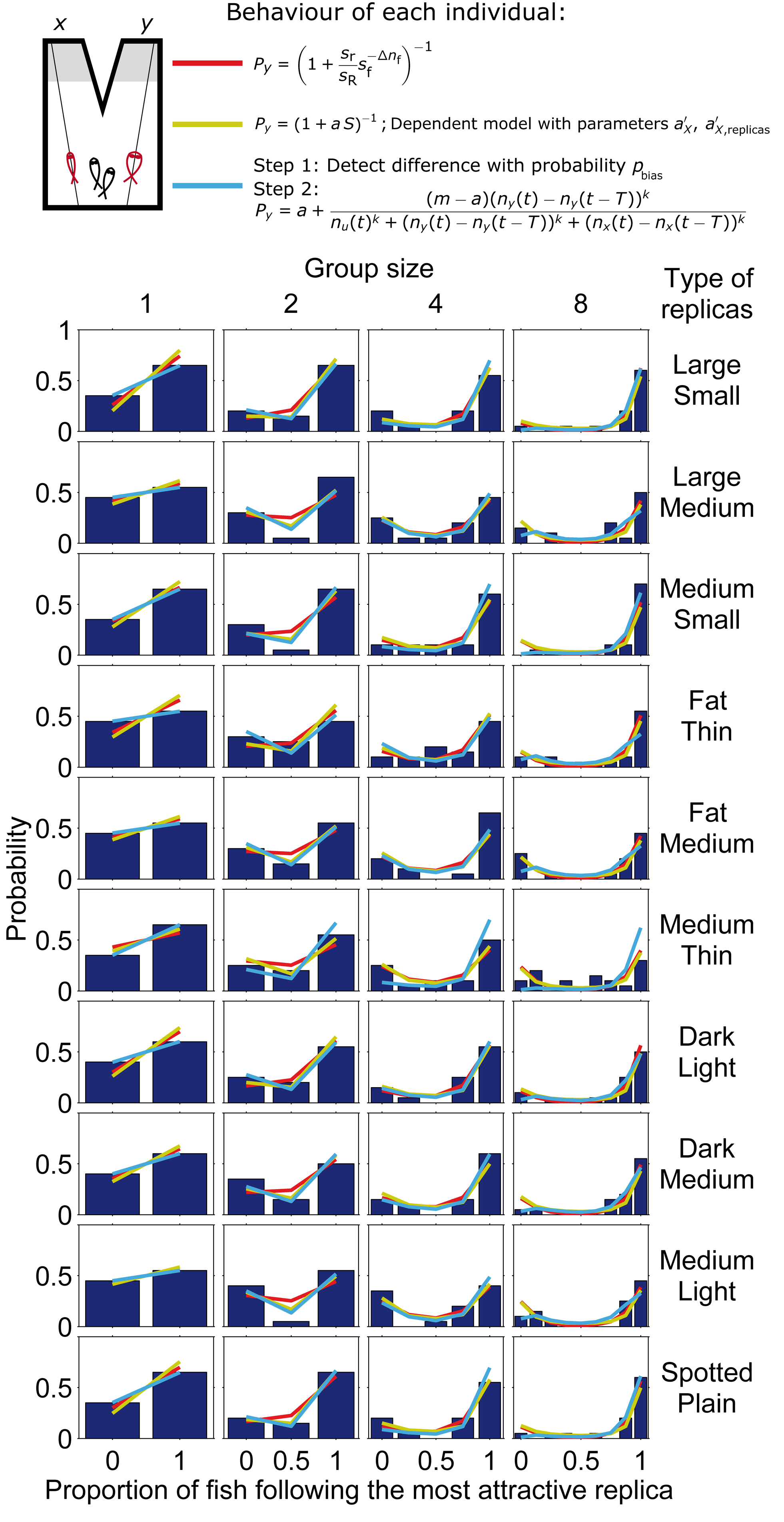

Supplement: Figure S2 — Comparison between different models for the condition with two different replicas. Experimentally measured statistics of final configurations of fish choices from 20 experimental repetitions [43] (blue histograms). Red line: results from model in Eq. 20 in the main text (, = 0.35, 0.7, 0.5, 0.52, 0.69, 0.75, 0.43, 0.55, 0.78, 0.43 for each row from top to bottom). Yellow line: Model including dependencies (, = 21.4, 11.8, 0.6, 9.9, 4.8, 0.9, 13, 8, 0.7, 14.5, 0.9 for each type of replica (large, medium, small, etc.). Blue line: Empirical model presented in Ref. [43], using the parameters reported there. Different graphs correspond to different stickleback group sizes and different types of replicas going to and . According to Bayesian Information Criterion (BIC, see Methods), our model neglecting dependencies gives the best representation of the data (red line, logprob , and BIC weight ). Second-best is out model including dependencies, (, ). Last, but near the second one, is the model from ref. [43] (blue line, . For the model from Ref. [43], these values of and correspond to a re-optimization of the model as described in Methods, because using the parameters reported in [43] would perform worse). The values of logprob () reported here do not include the data of the single-individual experiments (see Methods). (TIF) [file pcbi.1002282.s002.tif]

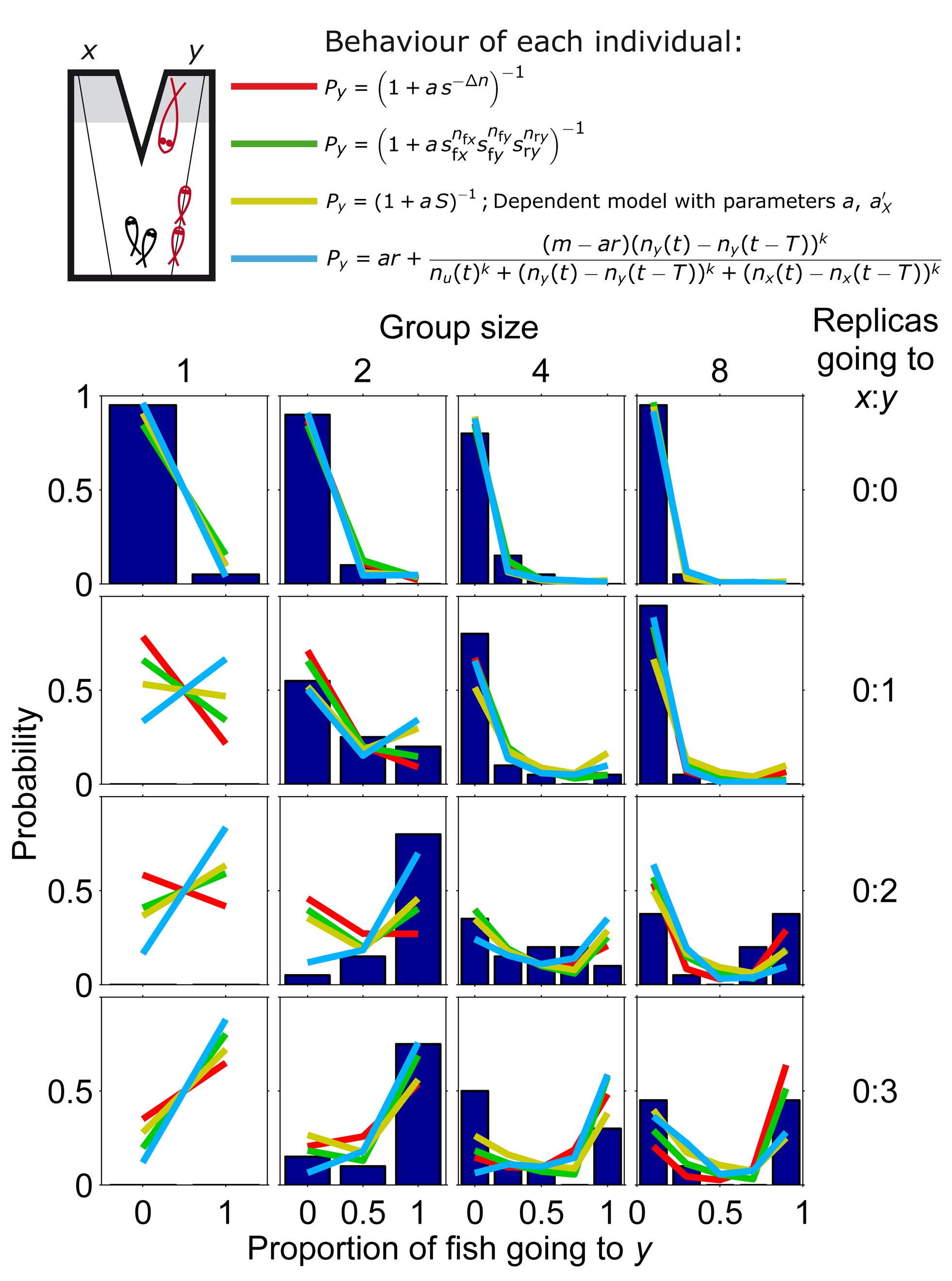

Supplement: Figure S3 — Comparison between different models in the asymmetrical set-up. Experimentally measured statistics of final configurations of fish choices from 20 experimental repetitions [42] (blue histograms). Red line: results from model neglecting dependencies in Eq. 22 in the main text (, ). Green line: Enhanced model neglecting dependencies with different reliability for the fish going to different locations and for the replicas (, , , . has no effect because there are no replicas going to ). Yellow line: Two-parameter model including dependencies (, ). Blue line: Empirical model presented in Ref. [42], using the parameters reported there. Different graphs correspond to different stickleback group sizes and different number of replicas going to . According to Bayesian Information Criterion (BIC, see Methods), the best two models are our complicated version neglecting dependencies (green line, logprob , and BIC weight ) and our two-parameter model including dependencies (yellow line, , ). Next (but very near) is our simplified model (red line, , ). And last (and significantly worse) the model from Ref. [42] (blue line, . For the model from Ref. [42], the values of and correspond to a re-optimization of the model as described in Methods, because using the parameters reported in [42] would perform worse. In two of the graphs for group size 1 that there are no data the prediction of the model from Ref. [42] and our model (especially the simplest version) are opposite. It might be that the results changed completely, depending on the results of these graphs, were the experiments performed. But we found that this is not the case: We performed simulations, adding experimental data in these two graphs. Even in the extreme case that the fabricated results matched exactly the predictions of the model in Ref. [42], BIC would still favour two of our models (we would get , for our model with dependence, , for our complicated model neglecting dependence, , for our simplified model neglecti [file pcbi.1002282.s003.tif]

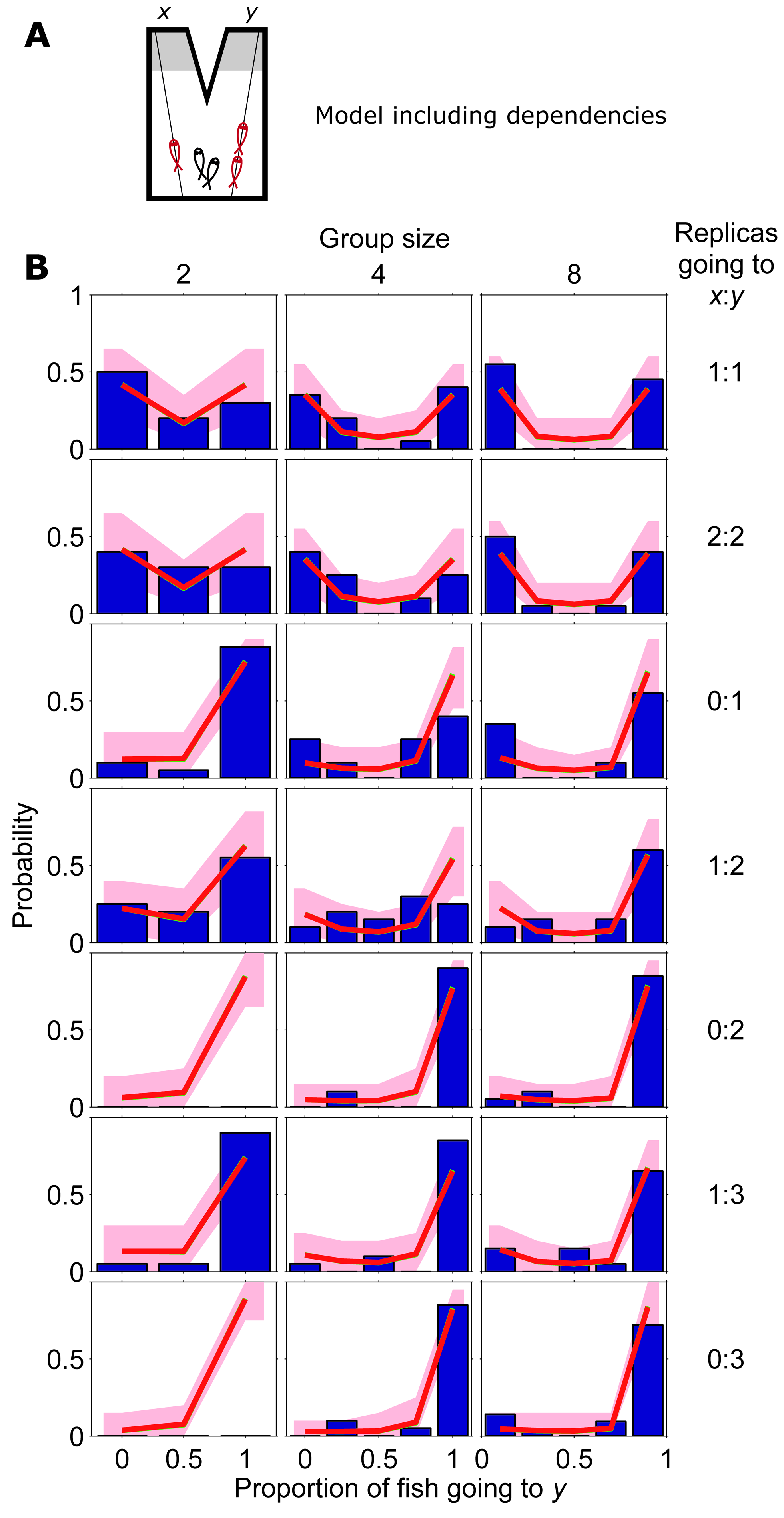

Supplement: Figure S4 — Comparison between model including dependencies and stickleback choices in symmetric set-up. (A) Schematic diagram of symmetric set-up with a group of sticklebacks (in black) choosing between two identical refugia and with different numbers of replica fish (in red) going to and . (B) Experimentally measured statistics of final configurations of fish choices from 20 experimental repetitions [42] (blue histogram) and results from the model that takes into account dependencies (red line using ; red region: 95% confidence interval; green line with ). Different graphs correspond to different stickleback group sizes and different number of replicas going to and . (TIF) [file pcbi.1002282.s004.tif]

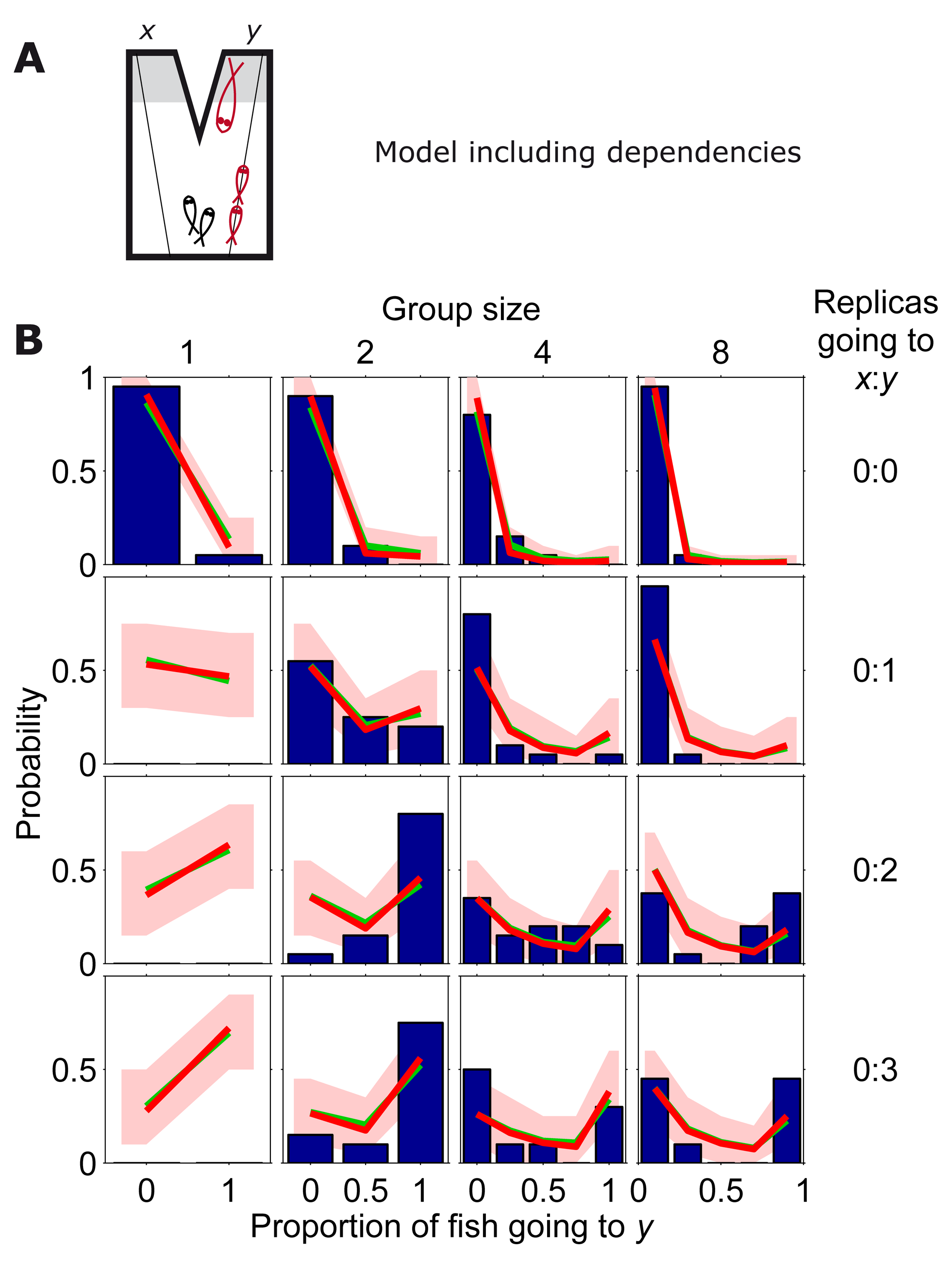

Supplement: Figure S5 — Comparison between model including dependencies and stickleback choices in asymmetric set-up. A) Schematic diagram of asymmetric set-up (predator at , large fish depicted in red) with a group of sticklebacks (in black) choosing between two refugia, and replica fish (small fish depicted in red) going to . (B) Experimentally measured statistics of final configurations of fish choices from 20 experimental repetitions [42] (blue histogram) and results from the model that takes into account the dependencies (red line using , ; red region: confidence interval. Green line using and ). Different graphs correspond to different stickleback group sizes and different number of replicas going to . (TIF) [file pcbi.1002282.s005.tif]

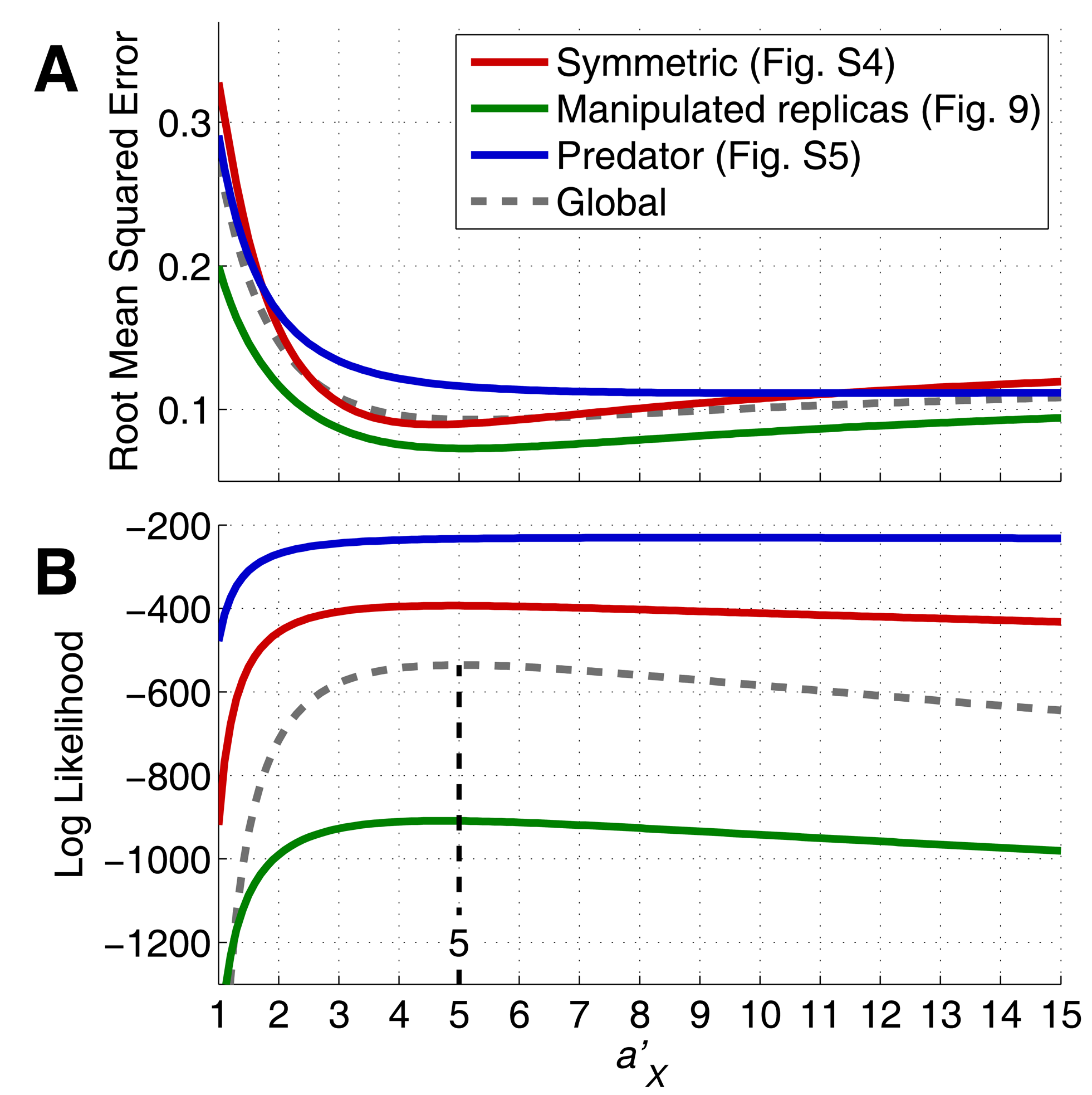

Supplement: Figure S6 — Goodness of fit of the model including dependencies for different values of . Red: Symmetric case (data in Fig. S4). Green: Case with different replicas at each side (data in Fig. 9. The parameters are re-optimized for each value of ). Blue: Asymmetric set-up with predator on one side (data in Fig. S5; Parameter is re-optimized for each value of ). (A) Root mean squared error between the data and the probabilities predicted by the model. Grey dashed line shows the mean RMSE for the three cases. The absolute values for each case depend on the shape of the data and are not comparable, only the trends and the position of the minima should be compared. (B) Logarithm of the probability that the data come from the model. The height of each curve depends on the number of data for each experiment, only the trend and the position of the maxima should be compared. Grey dashed line shows the sum of the three coloured lines, but shifted by 1000 so that it fits on the scale. The peak of this global probability indicates the value of that best fits the three datasets (). (TIF) [file pcbi.1002282.s006.tif]
