# Supplementary material for: Collective Animal Behavior from Bayesian Estimation and Probability Matching
Source: PLoS Comput Biol. 2011 Nov 17;7(11):e1002282. doi: 10.1371/journal.pcbi.1002282 (PMC3219619; doi:10.1371/journal.pcbi.1002282)
Supplement: Text S1 — Derivation of the model with more options. This file contains the derivation of the model for the more general case of different options (instead of only 2, as presented in the main text). (PDF) [file pcbi.1002282.s009.pdf]

Supporting Text

Animal collective behavior from Bayesian estimation and probability matching

Alfonso Pérez-Escudero[1,2,*], Gonzalo G. de Polavieja[1,2,*]

1 Instituto Cajal (CSIC) Madrid, Spain
2 Universidad Autónoma de Madrid, Spain
* E-mail: alfonso.perez.escudero@cajal.csic.es, gonzalo.polavieja@cajal.csic.es

Abstract

We present a derivation of the model for the more general case of M different options (instead of the 2 options used in the main text). We also discuss some particular cases that give simple expressions while still widely applicable.

Model for M options

Let M be the number of possible options, y_m, $m = 1 \dots M$. Each individual estimates the probability that each option is the best one, using its non-social information (C) and the behavior of the other individuals (B). So for one given option, say y_μ, we want to compute

$$P(Y_\mu|C,B), \tag{S1}$$

where Y_μ stands for 'y_μ is the best option'. We can compute the probability in Eq. **S1** using Bayes' theorem,

$$P(Y_\mu|C,B) = \frac{P(B|Y_\mu,C)P(Y_\mu|C)}{\sum_{m=1}^{M} P(B|Y_m,C)P(Y_m|C)}. \tag{S2}$$

Dividing numerator and denominator by the numerator, we get

$$P(Y_\mu|C,B) = \frac{1}{\sum_{m=1}^{M} a_{m\mu} S_{m\mu}}, \tag{S3}$$

where

$$a_{m\mu} = \frac{P(Y_m|C)}{P(Y_\mu|C)} \tag{S4}$$

contains only non-social information, and

$$S_{m\mu} = \frac{P(B|Y_m,C)}{P(B|Y_\mu,C)} \tag{S5}$$

contains the social information. Note that each term of the summation preserves the multiplicative relation between social and non-social information that was also apparent in Eq. 3 of the main text. There may be $M-1$ independent non-social parameters $a_{m\mu}$ in the case that no two options have equal non-social information. But usually this will not be the case, and the number of independent non-social parameters will be lower.

Now we assume independence among behaviors (Eq. 6 in main text), and group all possible behaviors in L classes, $\{\beta_k\}_{k=1}^{L}$ (Eq. 7 in main text). These two assumptions transform Eq. **S5** into

$$S_{m\mu} = \prod_{k=1}^{L} s_{k,m\mu}^{n_k}, \tag{S6}$$

where n_k is the number of individuals performing behavior β_k, and

$$s_{k,m\mu} = \frac{P(\beta_k|Y_m,C)}{P(\beta_k|Y_\mu,C)} \tag{S7}$$

are the reliability parameters for behavior β_k with respect to options y_m and y_μ. There may be up to $L(M-1)$ independent reliability parameters but usually they will not be all independent.

In summary, from Equations **S3** and **S6** we have that

$$P(Y_\mu|C,B) = \left(\sum_{m=1}^{M} a_{m\mu} \prod_{k=1}^{L} s_{k,m\mu}^{n_k} \right)^{-1}. \tag{S8}$$

This equation summarizes the general model applicable to any kind of experiment. In the following sections we consider two particular cases with a much simpler expression.

One basic reliability parameter

The general model in Eq. **S8** depends in general on $L(M-1)$ independent reliability parameters $s_{k,m\mu}$. Here we derive the model for a particular case in which there is only one reliability parameter, s.

First, we consider classes of behaviors (from now on we call them just 'behaviors') that simply consist of choosing a given option. If for example the options are different places, behaviors would be going to each of those places. Therefore, the number of possible behaviors is the same as the number of options, $L = M$. We use the convention that β_j is 'choosing option y_j'. Note that when a behavior is not informative (i.e. its reliability parameter is 1) it has no impact on the model in Eq. **S8**. Therefore, considering this set of behaviors is equivalent to assuming that all other behaviors have reliability parameter equal to 1.

We further assume that $P(\beta_k|Y_m,C)$ only depends on whether $k = m$ or $k \neq m$, so that

$$\begin{aligned} P(\beta_k|Y_k,C) &= P(\beta_l|Y_l,C) \\ P(\beta_k|Y_m,C) &= P(\beta_l|Y_p,C), \quad k \neq m,\ l \neq p \end{aligned} \tag{S9}$$

Note that $P(\beta_k|Y_k, C)$ is the probability that another individual makes the correct choice, and $P(\beta_k|Y_m, C) with\ k \neq m$ is the probability that it makes a wrong choice. So this assumption means that the probability of making the correct choice is the same regardless of which option is actually the correct one. In the case of symmetric choices, in which non-social information C is the same for all options, this relation will hold automatically, not being an extra assumption. It is likely that it also holds for many asymmetric choices. For example, the results for the asymmetric set-up presented in the main text suggest that it holds in that case. We define

$$\begin{aligned} p_c &\equiv P(\beta_k|Y_k, C) \\ p_f &\equiv P(\beta_k|Y_m, C), \quad k \neq m. \end{aligned} \tag{S10}$$

As it only matters whether the behavior matches the correct choice or not, there are only four distinct types of reliability parameters $s_{k,m\mu}$ (Eq. **S7**):

$$\begin{aligned} s_{k,kk} &= \frac{P(\beta_k|Y_k, C)}{P(\beta_k|Y_k, C)} = \frac{p_c}{p_c} = 1 \\ s_{k,ml} &= \frac{P(\beta_k|Y_m, C)}{P(\beta_k|Y_l, C)} = \frac{p_f}{p_f} = 1, \quad k \neq m,\ k \neq l \\ s_{k,km} &= \frac{P(\beta_k|Y_k, C)}{P(\beta_k|Y_m, C)} = \frac{p_c}{p_f} = s, \quad k \neq m \\ s_{k,mk} &= \frac{P(\beta_k|Y_m, C)}{P(\beta_k|Y_k, C)} = \frac{p_f}{p_c} = \frac{1}{s}, \quad k \neq m, \end{aligned} \tag{S11}$$

where

$$s \equiv \frac{p_c}{p_f} \tag{S12}$$

is the basic reliability parameter, equal to the probability that another individual makes the correct choice over the probability that it makes a mistake, for any behavior and for any individual. We regroup the terms inEq. **S8** so that it reflects the different types of $s_{k,m\mu}$ (Eq. **S11**), and get

$$P(Y_\mu|C, B) = \left(\sum_{m=1}^{M} a_{m\mu} s_{m,m\mu}^{n_m} s_{\mu,m\mu}^{n_\mu} \prod_{\substack{k=1 \\ k\neq m \\ k\neq\mu}}^{L} s_{k,m\mu}^{n_k} \right)^{-1}. \tag{S13}$$

Using the relations in Eq. **S11** we have that

$$P(Y_\mu|C, B) = \left(\sum_{m=1}^{M} a_{m\mu} s^{-(n_\mu - n_m)} \right)^{-1}. \tag{S14}$$

Note that the term $m = \mu$ is always equal to 1, so Eq. **S14** is identical to

$$P(Y_\mu|C, B) = \left(1 + \sum_{\substack{m=1 \\ m\neq\mu}}^{M} a_{m\mu} s^{-(n_\mu - n_m)} \right)^{-1}, \tag{S15}$$

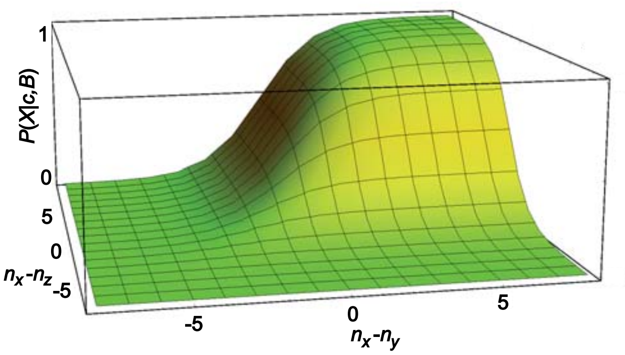

Figure 1. Probability of choosing one of the options for the 3-choice symmetric case.

that has the same structure as the equations presented in the main text.

Symmetric case

In the special case that all options are indistinguishable using non-social information alone (symmetric case), all non-social parameters $a_{m\mu}$ are equal 1 and Eq. **S15** becomes

$$P(Y_\mu|C, B) = \left(1 + \sum_{\substack{m=1 \\ m\neq\mu}}^{M} s^{-(n_\mu - n_m)} \right)^{-1}. \tag{S16}$$

We recall that in this case Eq. **S9** holds automatically, not being an extra assumption.

In the particular case of 3 options, x, y, z, we have

$$P(X|C, B) = \left(1 + s^{-(n_x - n_y)} + s^{-(n_x - n_z)} \right)^{-1}, \tag{S17}$$

and the corresponding expressions for $P(Y|C, B)$ and $P(Z|C, B)$. Fig. 1 shows $P(X|C, B)$ in terms of its two effective variables, $n_x - n_y$ and $n_x - n_z$ (Eq. **S17**).
